# Chrysin Modulates Aberrant Epigenetic Variations and Hampers Migratory Behavior of Human Cervical (HeLa) Cells

**DOI:** 10.3389/fgene.2021.768130

**Published:** 2022-01-12

**Authors:** Ritu Raina, Abdulmajeed G. Almutary, Sali Abubaker Bagabir, Nazia Afroze, Sharmila Fagoonee, Shafiul Haque, Arif Hussain

**Affiliations:** ^1^ School of Life Sciences, Manipal Academy of Higher Education, Dubai, United Arab Emirates; ^2^ Department of Medical Biotechnology, College of Applied Medical Sciences, Qassim University, Saudi Arabia; ^3^ Department of Medical Laboratory Technology, Faculty of Applied Medical Sciences, Jazan University, Jazan, Saudi Arabia; ^4^ Molecular Biotechnology Center, Institute of Biostructure and Bioimaging (CNR), Turin, Italy; ^5^ Research and Scientific Studies Unit, College of Nursing and Allied Health Sciences, Jazan University, Jazan, Saudi Arabia; ^6^ Bursa Uludağ University Faculty of Medicine, Görükle Campus, Bursa, Turkey

**Keywords:** phytochemicals, epigenetic modification, DNA methylation, epigenome, chrysin

## Abstract

**Purpose:** Plant-derived phytochemicals have shown epigenetic modulatory effect in different types of cancer by reversing the pattern of DNA methylation and chromatin modulation, thereby restoring the function of silenced tumor-suppressor genes. In the present study, attempts have been made to explore chrysin-mediated epigenetic alterations in HeLa cells.

**Methods:** Colony formation and migration assays followed by methylation-specific PCR for examining the methylation status of CpG promoters of various tumor-suppressor genes (TSGs) and the expression of these TSGs at the transcript and protein levels were performed. Furthermore, global DNA methylation; biochemical activities of DNA methyltransferases (DNMTs), histone methyl transferases (HMTs), histone deacetylases (HDACs), and histone acetyl transferases (HATs) along with the expression analysis of chromatin-modifying enzymes; and H3 and H4 histone modification marks analyses were performed after chrysin treatment.

**Results:** The experimental analyses revealed that chrysin treatment encourages cytostatic behavior as well as inhibits the migration capacity of HeLa cells in a time- and dose-dependent manner. Chrysin reduces the methylation of various tumor-suppressor genes, leading to their reactivation at mRNA and protein levels. The expression levels of various chromatin-modifying enzymes *viz* DNMTs, HMTs, HDACs, and HATS were found to be decreased, and H3 and H4 histone modification marks were modulated too. Also, reduced global DNA methylation was observed following the treatment of chrysin.

**Conclusion:** This study concludes that chrysin can be used as a potential epigenetic modifier for cancer treatment and warrants for further experimental validation.

## Introduction

Regular cell functions are modifiable by different epigenetic modifications, and these alterations play a crucial role during cellular growth and development (Rahman et al., 2016; Shankar et al., 2016). The epigenetic modifications occur by the way of regulatory mechanisms involving histone modifications, DNA methylation, microRNAs, and chromatin remodeling that modulates gene expression and disturbs cellular machinery and homeostasis in cancer cells (Huang et al., 2011; Ong et al., 2011; You and Jones, 2012; Ho et al., 2013; Aggarwal et al., 2015; Busch et al., 2015; Shankar et al., 2016). DNA methylation at CpG residues of the promoters of tumor suppressor genes (TSGs) causes repression of tumor-suppressor genes (Hatzimichael and Crook, 2013; Shankar et al., 2016), which is considered as a key regulatory mechanism of gene silencing and is correlated with the overexpression of DNA methyltransferases (DNMTs) (Kogan et al., 2017; Li and Wang, 2017; Piyathilake et al., 2017). Similarly, the modification of histone proteins by epigenetic enzymes, that is, HDAC (histone deacetylase), HMT (histone methyltransferase), HATs (histone acetyltransferase), and phosphorylases, leads to the repression or activation of gene activity. The equilibrium between the erasers and the writers of histone modifications is crucial for normal expression of genes, and their dysregulation may lead to cancer development (Sharma et al., 2009).

Methylation of histones at H3 and H4 lysine residues is catalyzed by histone methyl transferases (HMTs) (Ho et al., 2013; Shankar et al., 2016). Methylation marks at H3K79, H3K36, and H3K4 are supposed to be active marks, whereas H4K20, H3K27, and H3K9 methylation marks are related with transcriptional suppression (Sharma et al., 2009; Ho et al., 2013; Shankar et al., 2016). In the past, aberrant expression of both HMTs and HDMs has been reported in cancer (McLaughlin–Drubin et al., 2011; Busch et al., 2015). Histone acetylation is another important histone modification state, and hyper-acetylation leads to the stimulation of suppressed genes. Aberrant expression of HDAC has been found in cancer and is linked with gene repression and tumorigenesis (Sharma et al., 2009; Kogan et al., 2017; Andrijauskaite et al., 2019). Likewise, aberrant expressions of HATs and HDACs as well as HMTs and HDMs have been reported in various types of cancer in the past (Lin et al., 2009; Chen et al., 2017).

In the recent times, epigenetic-based cancer treatment is gaining more interest due to its reversible nature. Several FDA-approved drugs, for example, azacytidine and decitabine (DNMT inhibitors), and vorinostat and romidepsin (HDAC inhibitors), have shown promising results in solid malignancies and myelodysplastic syndrome (Herranz and Esteller, 2007a; Ong et al., 2011; Hatzimichael and Crook, 2013; Ho et al., 2013; Guo et al., 2015; Shankar et al., 2016). The combinational cancer treatment strategy in which both HDAC inhibitors and DNMT inhibitors are being used together has proven to be more effective (Herranz and Esteller, 2007a; Herranz and Esteller, 2007b; Ho et al., 2013; Shankar et al., 2016). However, low specificity and high systemic toxicity have limited their use (Paredes-Gonzalez et al., 2014). Hence, plant-derived chemopreventive agents are the main focus of scientific scope. Earlier studies have reported that dietary agents like EGCG, quercetin, genistein, curcumin, resveratrol, luteolin, and apigenin modulate the activity of DNMT and HDAC, and can lead to re-expression of silenced TSGs (Kai et al., 2010; Kim and Kim, 2013; Aggarwal et al., 2015; Guo et al., 2015; Mocanu et al., 2015; Chang and Yu, 2016a; Kanwal et al., 2016; Loh et al., 2019; Yan et al., 2020; Ganai et al., 2021a).

Chrysin (5-dihydroxyflavone), a flavone found in honey, bee propolis, and blue passion flower (*Passiflora caerulea*) extract, has gained importance as an antioxidant, antiviral, and anticancer compound (Pal-Bhadra et al., 2012; Yang et al., 2014; Kanwal et al., 2016). It induces cell cycle arrest, inhibits cell adhesion and tumor cell–induced angiogenesis, and induces apoptosis in various types of cancer, and also downregulates pathways including AKT (Khoo et al., 2010; Kasala et al., 2015; Ryu et al., 2017; Lim et al., 2018). Earlier, antiproliferative and apoptosis-inducing effects of chrysin on HeLa cells (Raina et al., 2021) have been observed. However, the role and mechanistic action of chrysin in the modulation of epigenome is not fully explored, except scanty reports wherein the role of chrysin in the modulation of epigenetic enzymes has been studied. Chrysin was found to decrease the expression of HDAC two and HDAC eight, and increase the expression of H4acK16, H3acK14, and H4acK12. It decreases H3me2K9 in (melanoma cell) A375 cells and restores the transcriptional activity of the tumor-suppressor gene *p21* ^*WAFI*^. Chrysin is capable enough to modify DNMT and HMT expressions in prostate cancer cells, and behaves as an epigenetic modifier (Pal-Bhadra et al., 2012; Kanwal et al., 2016; Ganai et al., 2021b). The precise mechanism of modulation of epigenome is not well explored and documented. Keeping the abovesaid facts in view, this study was performed to evaluate the significance of chrysin treatment on cell migration, DNA methylation, and histone modifications in human cervical cancer (HeLa) cells.

## Materials and Methods

### Maintenance of Cervical Cancer (HeLa) Cells and Drug Dilution

Human cervical cancer (HeLa) cells were used as an *in vitro* cancer model during this study. HeLa cells were maintained in complete Dulbecco’s modified Eagle medium (DMEM; Sigma-Aldrich; Merck, KGaA) containing 10% FBS (Sigma-Aldrich; Merck KGaA) and penicillin (100U/mL) (Sigma-Aldrich; Merck KGaA), and incubated at 37 °C with 5% CO_2_.

Chrysin (powdered, mol wt. 254.241 g/mol) was procured from Sigma-Aldrich (Merck, KGaA), and stock solution was prepared with DMSO (78.67 mM) using DMSO (stock solution). Furthermore, sub-stock (1 mM) and concentrations (5, 10, and 15 µM) of chrysin were prepared using the complete media as a diluent.

### Colony-Forming Assay

The colony-forming assay was performed following the protocol used by Crowley et al. (2016) and Sundaram et al. (2019), with minor modifications (Crowley et al., 2016; Kedhari Sundaram et al., 2019a). Briefly, **∼**2.5 × 10^5^ (Ho et al., 2013) cells were dispensed in six-well plates and incubated overnight followed by the treatment with increasing doses of chrysin (5, 10, and 15 µM) for 48 h. DMSO control (i.e., solvent control) and treated cells were collected after 48 h and plated at approximately 500 cells/well, and allowed to grow. The medium was changed time to time as per the requirement. After 14 days, the colonies formed were fixed by using absolute CH_3_OH and stained with crystal violet. Olympus inverted microscope (Labomed, United States) was used to obtain the images of the colonies formed. ImageJ software program was used to count the colonies in treated and DMSO control wells.

### Scratch-Wound Assay

The scratch-aound assay was performed to examine the inhibitory effect of chrysin on cell migration (Yang et al., 2014; Kedhari Sundaram et al., 2019a). Approximately 2 × 10^5^ (Ho et al., 2013) cells were plated in a six-well plate and incubated at 37°C overnight. On the next day, a “wound” or a “cell-free” line was created on a confluent monolayer of the cells by scratching the monolayer with a pipette tip. The cells were incubated in the presence of different chrysin dilutions (10 and 15 µM) and “healing” of wounds, which ensues through cell migration, and growth toward the cell-free zone was monitored on a regular basis. An inverted microscope was used for capturing the wound images in each well prior and after 24–48 h of the treatment.

### Trans-Well Chamber Assay

The invasion assay was performed to evaluate the migratory and invasive capability of chrysin-treated HeLa cells and DMSO control using the Boyden chamber (Yang et al., 2014). Briefly, 5.0 × 10^3^ (Aggarwal et al., 2015) cells/well were seeded on the upper side of the insert in separate inserts, and below it in the well of a 24-well plate, the medium with FBS was kept. After 48 h, absolute methanol was used to fix the cells, and 0.1% crystal violet was used for staining purposes. The cells present inside of the chamber were cleared using an ear bud. The assessment of complete migration was performed under the microscope, and any of the five fields were scanned (five fields per filter). The images were captured for each treatment and control using ×200 magnification with an inverted microscope (Olympus Corporation). ImageJ program was used for counting the colonies. The experiment was repeated three times, and mean ± SD was used to plot the graphs considering *p*-value ≤0.05.

### DNMT Activity Assay

Nuclear extracts from the untreated HeLa cells were prepared using the EpiQuik^TM^ Nuclear Extraction Kit (Catalog No. OP-0002, Epigentek, United States) following the manufacturer’s protocol. The EpiQuik DNMT Activity Assay Kit (Catalog No. #P-3009, Epigentek, USA) was used to check the effect of chrysin on the DNMT activity. Briefly, chrysin (@ conc. 5, 10 and 15 µM) was added to the untreated nuclear extract, buffer, and Adomet (methyl group donor) to the cytosine-rich DNA substrate–coated assay plate and incubated for 1.5 h at 37 °C. It was followed by incubation with capture and detection antibody. After signal development, the absorbance was read on an ELISA reader at 450 nm. DNMT activity inhibition was calculated by comparing with DMSO controls. The experiment was repeated three times, and the mean ± SD was used to plot the graph. The statistical significance level was calculated using one-way ANOVA, and the *p*-value was maintained at ≤ 0.05.

### HDAC Activity Assay

The EpiQuik HDAC Activity Assay Kit (Catalog Number P-4002, Epigentek, United States) was used for evaluating the effect of chrysin on the HDAC activity. The acetylated histone substrate–coated assay plate was prepared by adding 50 μL of the biotinylated HDAC substrate diluted in wash buffer to all wells. The assay plate was incubated at room temperature for 45 min and washed with wash buffer. HDAC assay buffer was dispensed to the wells in chrysin (5, 10, and 15 µM) treated and untreated nuclear extract placed. The plate was kept at 37°C for an hour followed by incubation with capture and detection antibody. Developing solution was used to develop the signal, stop solution stopped the reaction, and OD was read at 450 nm. The percentage inhibition of the HDAC activity against chrysin treatment was calculated by comparing with DMSO control. The experiment was repeated three times, and mean ± SD was used to plot a graph. One-way ANOVA was used to check the statistical significance at the *p*-value ≤ 0.05.

### HMT H3K9 Activity Assay

The assay was done by using the EpiQuik HMT H3K9 Activity Assay Kit (Catalog No. P-3003, Epigentek, USA) following the protocol given by the manufacturer. Briefly, to the histone three lysine substrate–coated assay plate, chrysin (@ conc. of 5, 10, and 15 µM) was added in separate wells, with untreated nuclear extract, buffer biotinylated substrate, and Adomet (methyl group donor), and incubated at 37 °C for 1.5 h. Following this step, capture antibody and detection antibody were added to the wells, and incubated at room temp for 30 min. Finally, a color was developed, and absorbance was measured using an ELISA reader at 450 nm. The percentage inhibition was calculated by comparing with DMSO control. The experiment was repeated three times, and the mean ± SD was calculated to plot a graph. One-way ANOVA was used for checking the statistical significance, and the *p*-value was maintained as ≤ 0.05.

### HAT Activity

The assay was done using the EpiQuik™ HAT Activity Assay Kit (Catalog No. P-4003 Epigenetek, United States) following the manufacturer’s protocol. Briefly, the nuclear extract and the substrate for HAT were incubated for 1 h followed by washing with the wash buffer. An inhibitor was added in the test samples, and signals were captured and detected by the capture and detection antibody, respectively. After color development, the plate was read at 450 nm using the ELISA plate reader. HAT activity and percentage inhibition were calculated by comparing with DMSO control samples. A graph was plotted by taking the mean of three experiments ± SD. One-way ANOVA was used to check the statistical significance at fixed *p*-value ≤ 0.05.

### Global DNA Methylation Assay

DNA isolation was done by using GenElute Mammalian Genomic DNA Miniprep Kit (Catalog No. G1N70, Sigma-Aldrich, Merck, KGaA) following the manufacturer’s protocol. About 1.5 × 10^6^ cells were treated with chrysin (@ conc. 5, 10, and 15 µM for 48 h) and DMSO control. DNA was isolated from chrysin treated and the DMSO control samples, and its quality was checked by gel electrophoresis using 1% agarose gel (Catalog No. A9539, Sigma-Aldrich, Merck, KGaA) in 0.5XTBE buffer with ethidium bromide. The quantitation of DNA samples was completed by spectrophotometry using NanoDrop 2000 (Thermo Scientific™, USA) and stored at-80°C.

MethylFlash™ Methylated DNA Quantification Kit (Catalog No. P-1034, Epigentek, USA) was used to analyze the methylated DNA in treated cells (@ chrysin conc. 5, 10, and 15 µm for 48 h) and DMSO controls. The kit was used for the detection of methylated DNA using antibodies against 5-mC (cytosine) that can be analyzed calorimetrically. Optical density was measured using the ELISA reader at 450 nm wavelength. As established, the extent of methylation on the gene is directly related to the optical density. The levels of methylation were calculated in comparison with the DMSO control. The experiment was performed three times at a significance level of *p*-value ≤0.05.

### Methylation-Specific PCR (MSRE–PCR)

CpG island DNA methylation quantification was conducted for estimating the percentage of methylated DNA in the total DNA content of HeLa cells by using the EpiTect Methyl II PCR System (Catalog No. 335452, Qiagen, United States). The method employed calculates the methylation of promoter regions in the input DNA after cleavage with methylation-dependent restriction enzymes and methylation-sensitive restriction enzymes that digest methylated and unmethylated DNA, respectively. Following restriction digestion, the cleaved DNA from each reaction was computed by using it as a template for Human Tumor Suppressor Genes EpiTect Methyl II Signature PCR Array (Qiagen, United States) real-time PCR in an assay plate with primers that border the promoter region of the anticipated genes. The relative amounts of unmethylated and methylated DNA were calculated by comparing the amounts of each reaction with that of a control (no enzymes added) reaction using the ^∆∆^CT method. The gene panel (with predesigned primers) consisted of tumor-suppressor genes comprising *TP73*, *MGMT*, *APC*, *CDKN2A*, *BRCA1*, *PTEN*, *CDH1*, *DAPK1*, *CDH13*, *SOC51*, *RARB*, *ESR1*, *FHIT*, *RASSF1*, *WIF1*, *GSTP1*, *RUNX3*, *MLH1*, *NEUROG1*, *VHL*, *PDLIM4*, and *TIMP3.*

The amount of DNA left after the restriction digestion was calculated by using qPCR array results. This was used for constructing the methylation profile of each gene with the ^∆Δ^CT method. The methylation and unmethylation fraction of the promoter of tested tumor-suppressor genes in chrysin-treated and untreated HeLa cells was estimated as per the protocol available with the kit. The levels of methylation were presented in the form of a graph. Statistical significance was calculated by taking the mean of three experiments by one-way ANOVA using the SPSS program with *p*-value *≤* 0.05.

### qRT‐PCR–Based Expression Analysis of Tumor-Suppressor, Migration, and Inflammation-Related Genes

The RNA from chrysin-treated (conc. 10 and 15 µM for 48 h) and DMSO control HeLa cells were extracted by using the GenElute Mammalian Genomic Total RNA Kit (Catalog No. RTN70 Sigma-Aldrich, Merck KGaA) and further quantified with the help of NanoDrop. The RNA (2 μg was used as a template) was then subjected to cDNA synthesis by using Applied Biosystems™ High-Capacity cDNA Reverse Transcription Kit (Catalog No. 4368814, ABI-Thermo Fisher, United States). This kit supports random primers’ scheme for initiating the synthesis of cDNA. The expression of genes related to various pathways of migration, inflammation, and TSGs was analyzed with the help of TaqMan-based custom array (4391524 and 4369514 master mix). The PCR array was run on QuantStudio3 and analyzed with the ^ΔΔ^ *C*_T_ method using the DataAssist™ program (Thermo Fisher, United States). GAPDH (housekeeping gene) was used for normalizing the data. Relative expression was calculated in comparison with the DMSO control. The statistical significance was calculated by maintaining *p*-value <0.05.

### Protein Expression by Proteome Profiler Array

The expression analysis of TSGs, migration, and inflammation-related proteins was performed by the Proteome Profiler Array (Catalog No. ARY026, R&D, USA). The relative expression levels of 84 oncogenes were investigated with the help of this array. Briefly, 1.5×10^6^ HeLa cells were plated in 25 cm^2^ flasks, and four such flasks were treated with 10 and 15 µM of chrysin for 48 h. The treated and DMSO control cells were collected and suspended in lysis buffer 17 (1 ml per 10^7^ cells) containing 10 μg/ml each of aprotinin (Catalog No. A6279; Sigma, USA), Leupeptin (Catalog No. 1167/25, Tocris, USA), and pepstatin (Catalog No. 1190/10, Tocris, United States) and shaken gently at 2–8°C for 30 min. The lysate produced was quantitated by the Pierce BCA Assay Kit (Catalog No. 23225; Thermo-Fisher Scientific, United States) following the manufacturer’s protocol. For this assay, 400 µg of a protein in 250 µL volume of the diluted cell lysate treated with chrysin (10 and 15 µM for 48 h) and the DMSO control was used for each membrane. The signal produced was then quantified by the chemiluminescent detector gel-doc system (Bio-Rad Laboratories, United States). The expression of proteins was analyzed by the intensity of proteins in the blot using Image Lab software (version 6.1). The fold change after normalization with the reference spot was calculated by comparing the treated (chrysin) values with the DMSO control values (mean of three experiments ±SD at *p*-value ≤ 0.05).

### Expression Analysis of Epigenetic Enzymes Involved in Chromatin Modification

Chromatin-modifying enzymes like writers—DNA and histone methyl transferases, histone acetyl transferases, and erasers like histone deacetylases and histone demethylases—help in dynamically sustaining cell metabolism and controlling processes such as cell growth propagation and gene expression by recognition of specific “marks” on histone proteins and DNA (Kouzarides, 2007). RNA was extracted from the DMSO control and chrysin-treated HeLa cells (10 and 15 µM for 48 h). RT^2^ Profiler™ PCR Array Human Epigenetic Chromatin Modification Enzymes (Catalog No. PAHS-085Z, Qiagen, United States) were used to check the expression of genes responsible for the modulation of DNA and histones including DNA methyl transferases, histone methyl transferases, histone acetyl transferases, and demethylases. RNA at a concentration of 1 µg was used to synthesize cDNA, and it was diluted to 1,350 µL with nuclease-free water and an equal amount of RT2 SYBR^®^ Green qPCR Master mix (Catalog No. 330504; Qiagen, United States) was added to this. From this mixture, 25 µL was poured into each well of the array plate having predefined primers, and the plate was run on ABI Quant Studio 3. The normalization was performed using GAPDH housekeeping gene and the fold change was calculated by comparing the chrysin-treated samples with the DMSO control. The statistical significance was calculated at *p*-value ≤ 0.5.

### H3 and H4 Histone Modification Marks

In order to understand the role of chrysin as an epigenetic modifier, the Histone Extraction kit (Catalog No. ab113476, Abcam, Cambridge, UK), and Histone H3 and H4 Modification Multiplex Assay kits (Catalog Nos. ab185910 and ab185914) were procured from Abcam, Cambridge, UK. Following the extraction of histone using the Histone Extraction Kit, ∼100 ng of histone protein was used per well, and the protocol given by the manufacturer was followed. OD was measured at 405 nm, and graphs were plotted for reflecting the effect of chrysin compared to the DMSO control. The experiments were performed in triplicates, one-way ANOVA was used to determine the significance of the experiments, and *p*-value was maintained at ≤0.05.

### Statistical Analysis

Statistical analysis was performed by SPSS program (version 21). The data were examined by using one-way ANOVA followed by Tukey’s HSD *post hoc* analysis. All experiments were performed in triplicate. The results are expressed as the mean ± SD of three distinct experimentations. The statistical significance was set at *p*-value ≤0.05.

## Results

### Chrysin-Inhibited Colony Formation and Migration of HeLa Cells

**FIGURE 1:**
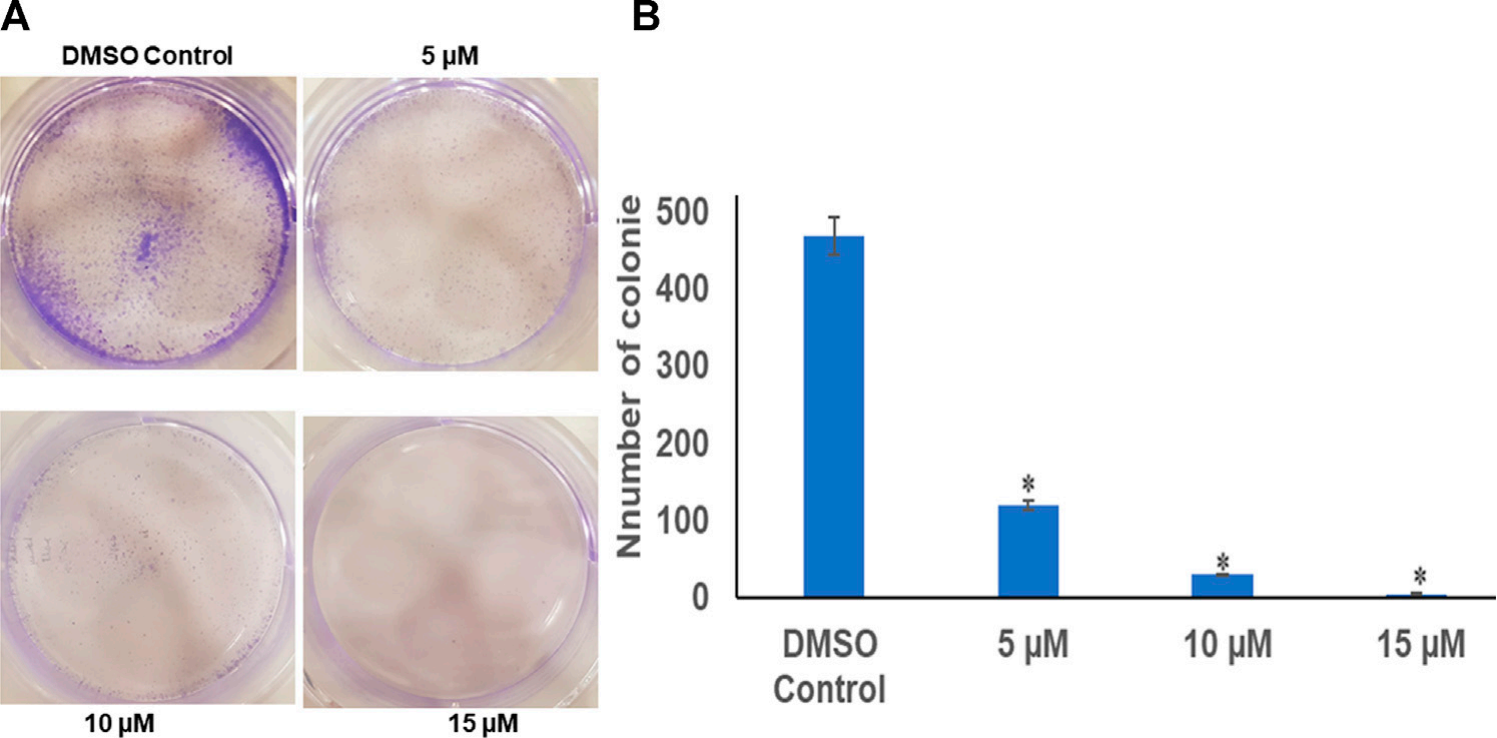
**(A)** Chrysin inhibits colony formation in a dose-dependent manner with almost no colonies at 15 µM chrysin treatment for 48 h. **(B)** Graphical representation of inhibition of colony formation.

The colony-forming assay was performed to understand the long-term effect of chrysin on the growth and division of HeLa cells. After calculating the survival factor (SF), it was observed that the DMSO control had plating efficiency (PE) of 95%, whereas the survival factor for 5 and 10 µM of chrysin showed only 120 and 30 colonies, respectively. At 15 µM chrysin conc., very few colonies were formed. Hence, it can be inferred that chrysin restrained the capability of cells to form colonies. These results suggest that chrysin is not only capable of causing cell death but also leads to cytostatic state (Figure 1A and Figure 1B).

**FIGURE 2:**
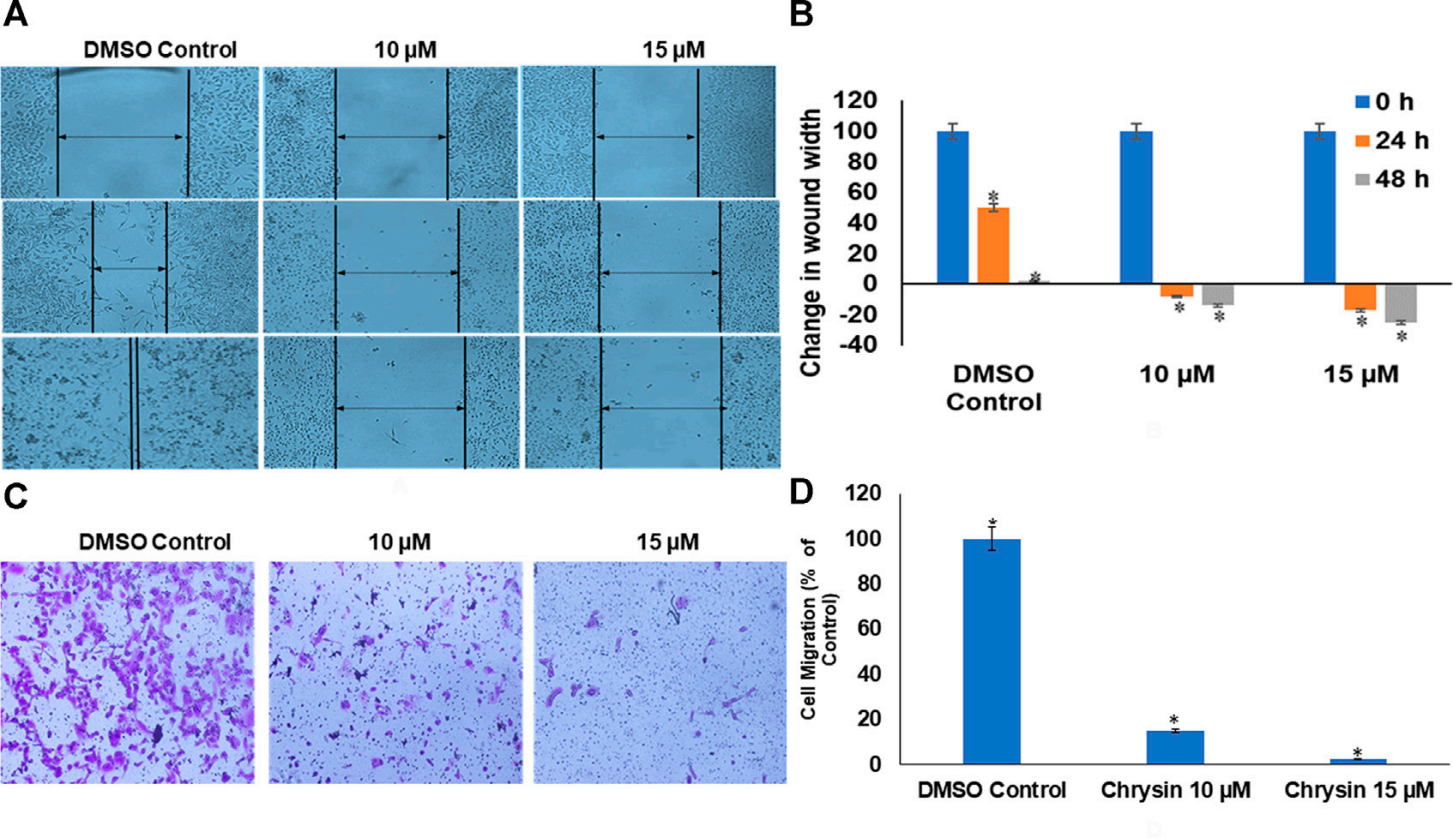
**(A)** Chrysin treatment prevented migration of HeLa cells, as compared to the DMSO control wells, the chrysin treatment showed increase in wound width after 10–15 µM treatment. **(B)** Graphical representation of increase in wound width after chrysin treatment at 10–15 µM concentrations for 24–48 h. **(C)** The chrysin-treated HeLa cells depicted significant decrease in cell migration using trans-well inserts. **(D)** Graphical representation of inhibition of cell migration by chrysin.

Likewise, chrysin decreased the migration capacity of HeLa cells as demonstrated by scratch-wound and invasion assay using trans-well. Chrysin increased the wound width by 8 and 14% at 10 µM conc. treatment for 24 and 48 h and 17 and 25% at 15 µM conc. treatment for 24 and 48 h, respectively, whereas in DMSO control cells, there was almost complete wound closure after 48 h (Figure 2A and Figure 2B). This was further corroborated by significant decrease in the number of migrating cells after chrysin treatment using the trans-well assay. Only 15 and 2.5% migration at 10 and 15 µM chrysin treatment for 48 h was observed, respectively, in comparison with the DMSO control cells (Figure 2C and Figure 2D).

### Chrysin Reexpresses Tumor-Suppressor Genes (TSGs) and Downregulates Genes Related to Migration and Inflammation

**FIGURE 3:**
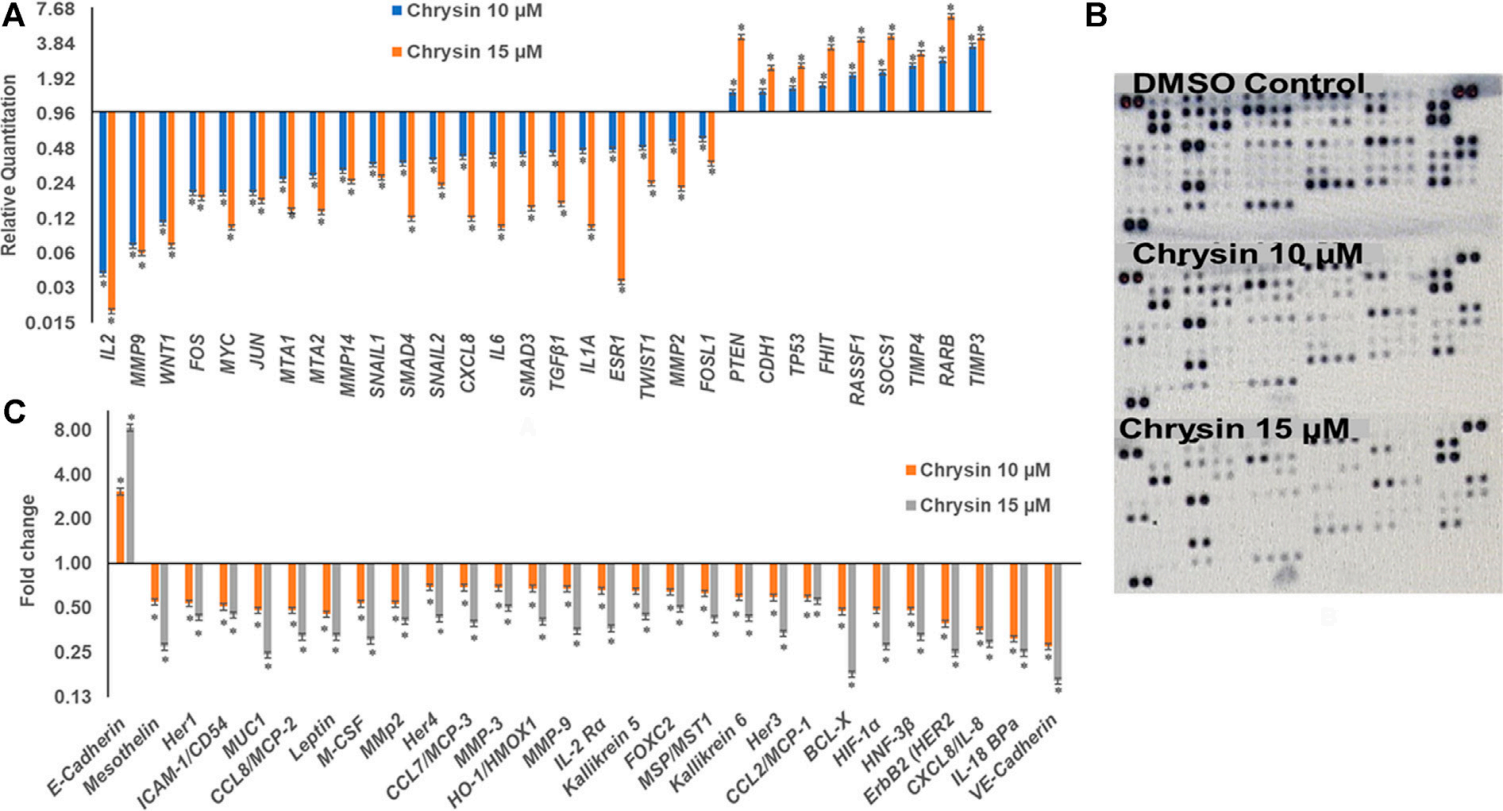
**(A)** Chrysin modulated the expression of various TSGs and migration related genes in a dose-dependent manner. The TSGs were reactivated, whereas inflammatory- and migration-related genes were downregulated. **(B)** The nitrocellulose membranes depicting the expression of different proteins. **(C)** Chrysin treatment modulates the proteins related to migration and inflammation in a dose-dependent manner.

qPCR was done to understand the effect of decreased methylation of various tumor-suppressor genes following the treatment of chrysin. It was observed that chrysin treatment increased the expression of various TSGs (such as *TIMP3*, *TIMP4*, *RARB*, *RASIF1*, *TP53*, *PTEN*, *CDH1*, and *SOCS1*) and reduced the expression of genes responsible for metastasis (*viz. MMP 2*, *MMP 9*, *MMP 14*, *SNAIL1*, *SMAD3*, *SMAD4*, and *MTA1*, *2*) and the genes involved in the inflammatory process (*viz. IL2*, *IL1A*, *IL6*, and *CxCL8*). Chrysin treatment also decreased the expression of oncogenes like *FOS*, *JUN*, *MYC*, *ESR1*, and *TWIST1* (Figure 3A and Table 1). Relative quantification (RQ) derived from the 2^–∆∆Ct^ method specifies the fold change in gene expression against the DMSO control after normalization with the selected endogenous gene (GAPDH). The upregulation is documented at RQ ≥ 1.5 and downregulation at RQ ≤ 0.5.

**TABLE 1:** Relative expression of TSGs and genes related to migration and metastasis. The values are taken as mean of three experiments ±SD (*p* ≤ 0.05).

Genes	Gene ensemble no.	Gene information	RQ chrysin 10 µM	RQ chrysin 15 µM
IL2	Hs00174114_m1	Interleukin 2	0.04	0.02
MMP9	Hs00234579_m1	Matrix metallopeptidase 9	0.07	0.06
WNT1	Hs00180529_m1	Wnt family member 1	0.11	0.07
FOS	Hs00170630_m1	Fos proto-oncogene	0.20	0.18
MYC	Hs99999003_m1	Myelocytomatosis viral oncogene homolog	0.20	0.10
JUN	Hs99999141_s1	Jun proto-oncogene	0.20	0.17
MTA1	Hs00183042_m1	Metastasis associated 1	0.26	0.14
MTA2	Hs00191018_m1	metastasis associated 2	0.28	0.14
MMP14	Hs01037009_g1	matrix metallopeptidase 14	0.31	0.25
SNAIL1	Hs00195591_m1	Snail family transcriptional repressor1	0.35	0.27
SMAD4	Hs00232068_m1	SMAD family member 4	0.36	0.12
SNAIL2	Hs00161904_m1	Snail family transcriptional repressor2	0.38	0.23
CXCL8	Hs99999034_m1	C-X-C motif chemokine ligand 8	0.41	0.12
IL6	Hs00174131_m1	Interleukin 6	0.42	0.10
SMAD3	Hs00969210_m1	SMAD family member 3	0.43	0.15
*TGFβ1*	Hs00998133_m1	Transforming growth factor beta 1	0.44	0.16
*IL1A*	Hs00174092_m1	Interleukin 1 alpha	0.46	0.10
*ESR1*	Hs01046816_m1	Estrogen receptor 1 metastasis associated 2	0.47	0.03
*TWIST1*	Hs00361186_m1	Twist family bHLH transcription factor 1	0.49	0.24
*MMP2*	Hs00234422_m1	Matrix metallopeptidase 2	0.55	0.22
*FOSL1*	Hs04187685_m1	1FOS-like 1	0.58	0.36
*PTEN*	Hs01920652_s1	Phosphatase and tensin homolog	1.48	4.41
*CDH1*	Hs00170423_m1	Cadherin 1	1.50	2.40
*TP53*	Hs01034249_m1	Tumor protein p53	1.60	2.50
*FHIT*	Hs00896860_m1	Fragile histidine triad	1.70	3.60
*RASSF1*	Hs00945255_g1	Ras association domain family member 1	2.08	4.20
*SOCS1*	Hs00705164_s1	Suppressor of cytokine signaling 1	2.20	4.50
*TIMP4*	Hs00162784_m1	TIMP metallopeptidase inhibitor 4	2.50	3.20
*RARB*	Hs00977140_m1	Retinoic acid receptor beta	2.80	6.70
*TIMP3*	Hs00165949_m1	TIMP metallopeptidase inhibitor 3	3.70	4.40

### Chrysin Modulates the Protein Expression of Genes Involved in Migration, Inflammation, and Tumor Suppression

Proteome profiler–based quantitation of proteins that are involved in proliferation and migration and other cellular events revealed chrysin-supported modulation was consistent with mRNA expression. The treatment of HeLa cells with 10 and 15 µM of chrysin resulted in the downregulation of the expression of various proteins related to migration *viz* MMP2, MMP9, and MMP3, mesothelin, MUC1, leptin, and M_CSF; inflammatory proteins like CCL8/MCP-2, CCL7/MCP-3, IL-18 BPa, CXCL8/IL-8, and IL-2 Rα; and oncogenes like HER1, 2, 3, and 4, and ICAM-1/CD54. Proteins related to cell proliferation, growth, and apoptosis like BCL-X, HIF-1α, HNF-3β, and HO-1/HMOX1 were also downregulated, that is, related to tumor progression, whereas upregulation of E-cadherin (CDH1) was observed after chrysin treatment (Figure 3B, Figure 3C and Table 2). Fold changes in protein expression were calculated by comparing the treated cells with those of the DMSO control. The upregulation was fixed at ≥1.5 fold and downregulation at ≤0.5 fold.

**TABLE 2:** Expression of proteins involved in migration and inflammation. The values are taken as mean of three experiments ±SD (*p* ≤ 0.05).

Oncogene proteins	Fold change chrysin 10 µM	Fold change chrysin 15 µM
E-cadherin	3.07	8.27
Mesothelin	0.55	0.27
Her1	0.54	0.43
ICAM-1/CD54	0.51	0.45
MUC1	0.48	0.24
CCL8/MCP-2	0.48	0.32
Leptin	0.46	0.32
M-CSF	0.53	0.30
MMp2	0.53	0.41
Her4	0.69	0.43
CCL7/MCP-3	0.69	0.39
MMP-3	0.69	0.50
HO-1/HMOX1	0.68	0.41
MMP-9	0.67	0.35
IL-2 Rα	0.66	0.36
Kallikrein 5	0.65	0.44
FOXC2	0.65	0.50
MSP/MST1	0.63	0.42
Kallikrein 6	0.59	0.43
Her3	0.59	0.34
CCL2/MCP-1	0.59	0.56
BCL-X	0.48	0.18
HIF-1*α*	0.48	0.28
HNF-3*β*	0.48	0.32
ErbB2 (HER2)	0.39	0.25
CXCL8/IL-8	0.35	0.29
IL-18 BPa	0.31	0.25
VE-cadherin	0.28	0.16

### Chrysin Inhibits DNMT Activity in HeLa Cells

**FIGURE 4:**
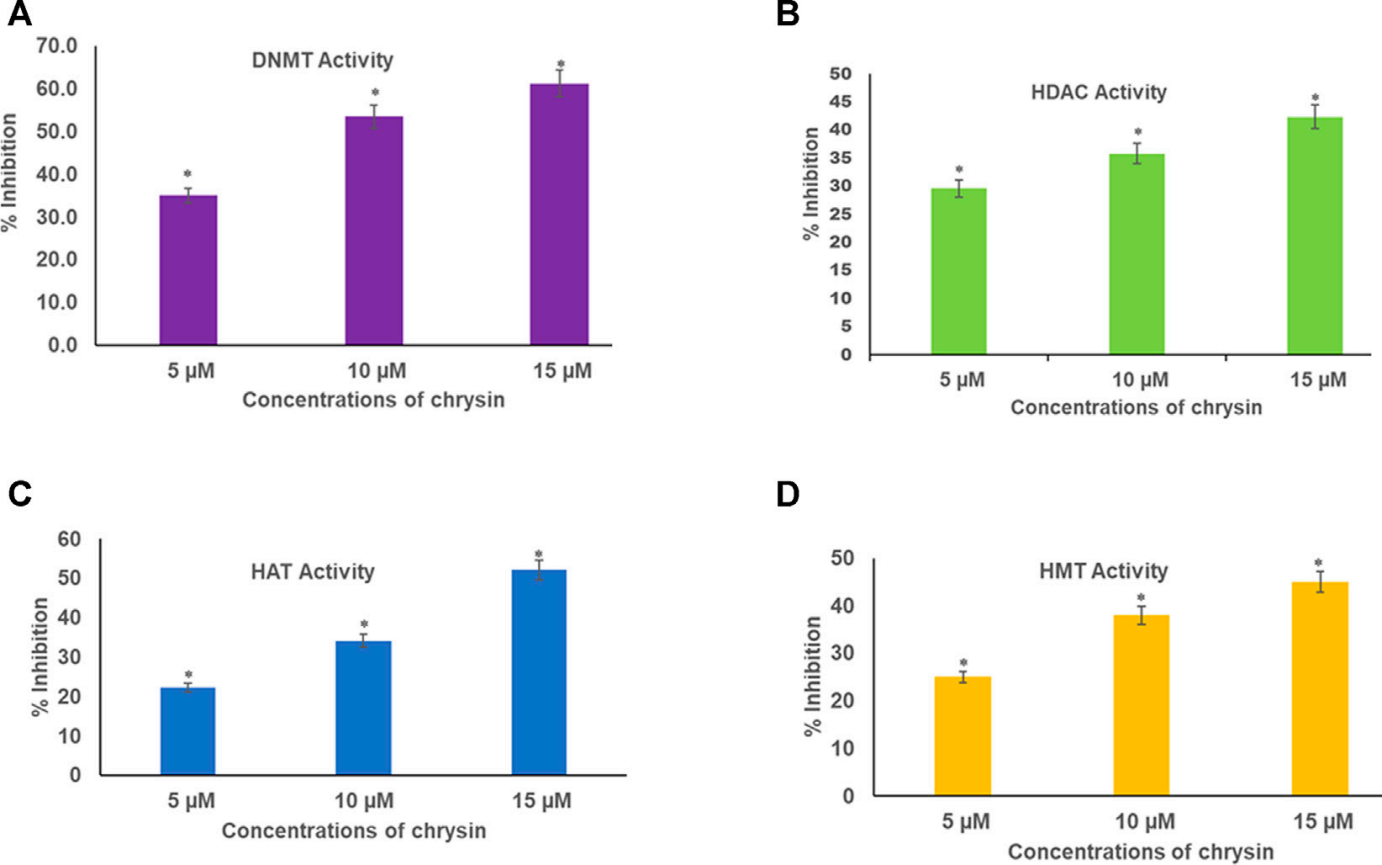
**(A)** Chrysin decreased DNMT activity in HeLa cells irrespective of their location on the genome in a concentration-dependent manner. **(B)** Chrysin inhibited HDAC activity in HeLa cells in a concentration-dependent manner. The activity decreased with increase in the dose of chrysin **(C)** Chrysin decreased the activity of HAT in a dose-dependent manner. As the concentration of chrysin increased, the inhibition percentage increased. **(D)** Chrysin decreased HMT H3K9 enzyme activity in HeLa cells, irrespective of their location on the genome in a concentration-dependent way.

Chrysin inhibited DNMT activity in HeLa cells in a dose-dependent manner. The incubation of the nuclear extract with 5, 10, and 15 µM of chrysin resulted in the inhibition of DNMT activity by 35, 54, and 61%, respectively, compared to the DMSO control (Figure 4A).

### Chrysin Inhibits HDAC Activity

Nuclear extracts were incubated with increasing concentrations (5, 10, and 15 µM) of chrysin; it was found that it inhibited the activity of HDACs by 30, 36, and 42% in a dose-dependent response compared with the DMSO control (Figure 4B).

### Chrysin Decreases HAT Activity in a Dose-Dependent Manner

Histone acetyl transferases cause acetylation at N-terminal tails of histone proteins. The incubation of nuclear extract with varying concentrations of chrysin (5, 10, and 15 µM) showed decline in HAT activity in the treated cells compared to the DMSO control. A decrease of 22, 34, and 52% was observed at 5, 10, and 15 µM conc. of chrysin treatment, respectively (Figure 4C).

### Chrysin Reduces HMT H3K9 Enzyme

HMT H3K9 can add methyl groups at histone three and lysine 9. All the methylation marks—mono, di, and trimethylation—are repressive marks. The incubation of HeLa cell nuclear extracts with 5, 10, and 15 µM conc. of chrysin reduced the activity of the enzyme by 25, 38, and 45%, respectively (Figure 4D).

### Chrysin Modifies the Expression of Chromatin-Modifying Genes

**FIGURE 5:**
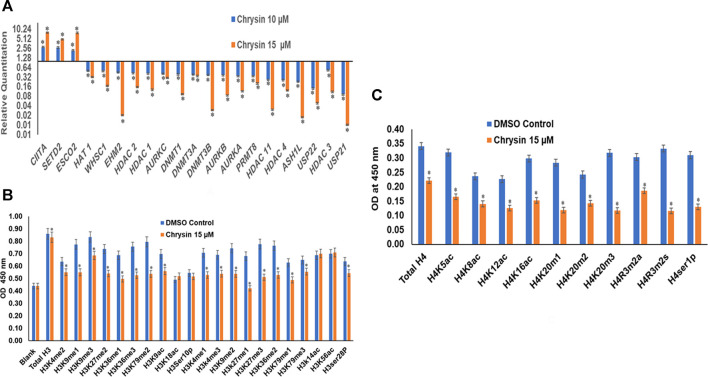
**(A)** Treatment of HeLa cells with chrysin at 10 and 15 µM for 48 h modulated the expression of epigenetic enzymes (HDACs, DNMTs, HATs, HMTs *etc*.) in a dose-dependent manner. **(B)** Chrysin modulates the H3 acetylation and methylation histone marks at 15–48 h. **(C)** H4 histone marks modulated by chrysin as compared to the DMSO controls.

RT (Rahman et al., 2016) Profiler™ PCR Array Human Epigenetic Chromatin Modification Enzymes (Catalog No. PAHS-085Z; Qiagen, USA) were used to check the expression of various chromatin-modifying enzymes following the treatment of chrysin (@ conc. 10 and 15 µM) for 48 h compared to the DMSO control. Chrysin treatment down-regulated the expression of DNA methyltransferases like *DNMT1*, *3A*, and *3B* significantly. *HDAC1*, *2*, *3*, *4*, and *11* also showed a steep decline after the above-stated chrysin treatment. Remarkably, downregulation of *WHSC1*, *AURKA*, *AURKB*, and *AURKX*. *EHM2*, *PRMT8*, and *HAT1* were also observed after 10 and 15 µM of chrysin treatment. However, enhanced expression of *SETD2*, *ESC O 2*, and *CIITA* was found after the same chrysin treatment (RQ in Figure 5A and Table 3).

**TABLE 3:** RQ values of chromatin-modifying enzymes after chrysin treatment. The values are taken as mean of three experiments ±SD (*p* ≤ 0.05).

Gene information	Genes	RQ chrysin 10 µM	RQ chrysin 15 µM
Class II major histocompatibility complex transactivator	CIITA	2.80	7.90
SET domain containing	SETD2	2.70	4.90
Establishment of sister chromatid cohesion N-acetyltransferase 2	ESCO2	2.20	7.80
Histone deacetylase IV	HAT 1	0.50	0.32
Wolf–Hirschhorn syndrome candidate 1 gene	WHSC1	0.49	0.17
A member of NF2/ERM/4.1 superfamily	EHM2	0.44	0.02
Histone deacetylase 2	HDAC 2	0.42	0.15
Histone deacetylase 1	HDAC 1	0.41	0.13
Aurora kinase C	AURKC	0.40	0.30
DNA methyl transferase 1	DNMT1	0.39	0.09
DNA methyl transferase 3A	DNMT3A	0.38	0.34
DNA methyl transferase 3B	DNMT3B	0.37	0.03
Aurora kinase B	AURKB	0.36	0.08
Aurora kinase A	AURKA	0.34	0.11
Protein Arginine methyltransferase 8	PRMT8	0.34	0.20
Histone deacetylase 11	HDAC 11	0.26	0.03
Histone deacetylase 4	HDAC 4	0.25	0.12
A SH1-like histone lysine methyltransferase	ASH1L	0.23	0.02
Ubiquitin-specific peptidase 22	USP22	0.14	0.05
Histone deacetylase 3	HDAC 3	0.52	0.11
Ubiquitin-specific peptidase 21	USP21	0.09	0.01

### Chrysin Modulates H3 and H4 Histone Marks

Chrysin modulated the expression of methylation, acetylation, and phosphorylation H3 and H4 marks. H3K9me1, H3K9me2, H3K9me3, H3K27me1, H3K27me2, H3K27me3, H3K36me1, H3K36me3, H3K79me1, H3K79me2, and H3K79me3 marks were reduced after the treatment of HeLa cells with 15 µM of chrysin for 48 h; similarly, H3 acetylation marks were diminished after treatment with 15 µM of chrysin (Figure 5B). The expression of H3K9ac, H3K18ac, H3K14ac, and H3K56ac was reduced after chrysin treatment. Likewise, the acetylation marks at H4 were also modulated against chrysin treatment including H4K5ac, H4K8ac, H4K12ac, and H4K16ac. H4 methylation marks, like H4K20me1, H4K20me2, and H4K20me3, showed decreased expression after chrysin treatment (Figure 5C). Phosphorylation marks of H3ser28p, H4ser10 p, H4R3m2a, and H4Rm2s were also decreasingly expressed after 15 µM chrysin treatment of HeLa cells for 48 h (Figure 5B and 5C).

### Chrysin Diminishes Global DNA Methylation of HeLa Cells

**FIGURE 6:**
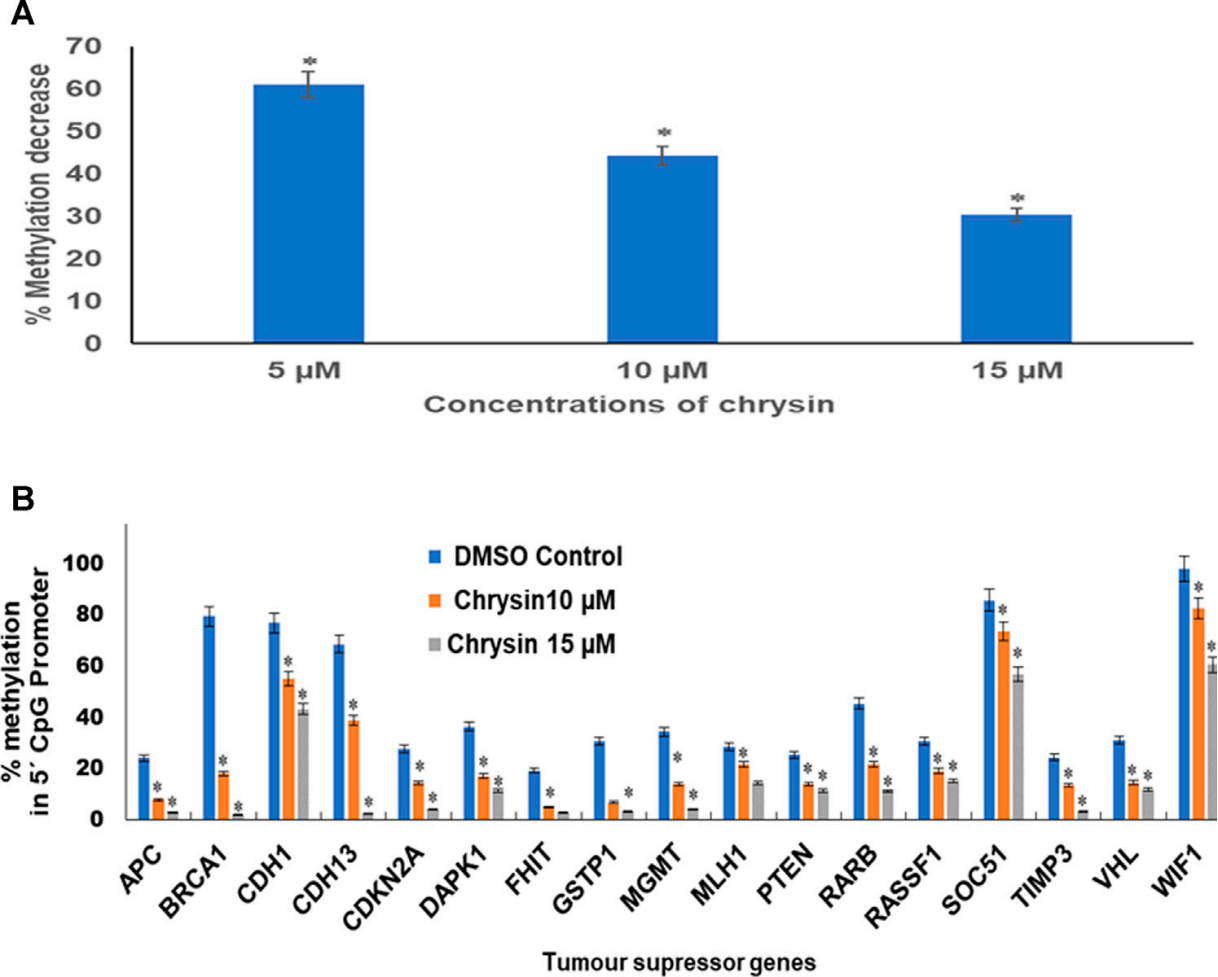
**(A)** Chrysin treatment at 5, 10, and 15 µM–48 h decreased the global DNA methylation in a dose-dependent manner. **(B)** Chrysin treatment of HeLa cells at 10 and 15 µM for 48 h demonstrated profound decrease in percent methylation in 5′ CpG promoter regions of TSGs as compared to the DMSO controls in a dose-dependent manner.

An obvious decrease in global methylation was observed after 48 h treatment of 5, 10, and 15 µM of chrysin against HeLa cells. Global DNA methylation was studied by comparing with the DMSO control. This was reduced to 61, 44, and 30% against 5, 10, and 15 µM chrysin treatment of HeLa cells, respectively (Figure 6A).

### Chrysin Reduces Methylation of the Promoter Region of Various Tumor-Suppressor Genes

Methylation-sensitive restriction enzyme PCR revealed that chrysin decreased the promoter methylation of crucial tumor-suppressor genes of HeLa cells. These TSGs included *APC*, *BRCA1*, *CDH1*, *PTEN*, *GSTP1*, *FHIT*, *DAPK1*, *CDH13*, *CDKN2A*, *MGMT*, *MLH1*, *RARB*, *RASSF1*, *SOCS1*, *VHL*, *WIFI*, and *TIMP3*. The methylation percentage of the abovesaid genes decreased significantly as *APC* (8%, 3%), *BRCA1* (18%, 2%), *CDH1* (55%, 43%), *CDH13* (39%, 3%), *CDKN2A* (14%, 4%), *DAPK1* (17%, 11%), *FHIT* (5%, 3%), *GSTP1* (7%, 3%), *MGMT* (14%, 4%) *MLH1* (22%, 15%), *PTEN* (14%, 11%), *RARB* (22%, 11%), *RASSF1* (19%, 15%), *SOCS1* (73%, 56%), *TIMP3* (13%, 3%), *VHL* (15%, 12%), and *WIFI* (82%, 61%) at 10 and 15 µM chrysin, respectively, compared to the DMSO control, wherein the methylation percentage was much higher (Figure 6B and Table 4).

**TABLE 4:** Percentage of CpG promoter methylation after chrysin treatment as compared to untreated control. The values are taken as mean of three experiments ±SD (*p* ≤ 0.05).

Target name	Gene name	Untreated control	Chrysin 10 µM	Chrysin 15 µM
APC	Adenomatous polyposis 1	24.20	7.88	2.72
BRCA1	Breast cancer gene 1	79.49	18.12	2.11
CDH1	E-cadherin	76.91	55.01	43.25
CDH13	H-cadherin	68.56	38.89	2.60
CDKN2A	Cyclin-dependent inhibitor 2A	27.74	14.43	4.19
DAPK1	Death-associated protein kinase 1	36.49	17.06	11.46
FHIT	Fragile histidine triad protein	19.35	5.04	2.94
GSTP1	Glutathione S-transferase Pi 1	30.80	6.88	3.11
MGMT	O-6-Methylguanine-dna methyltransferase	34.38	13.99	4.29
MLH1	Mutl homolog 1	28.49	21.88	14.50
PTEN	Phosphatase and tensin homolog	25.43	14.11	11.30
RARB	Retinoic acid receptor beta	45.47	21.88	11.22
RASSF1	Ras association domain family member 1	30.71	19.24	15.27
SOC51	Suppressor of cytokine signaling 1	85.82	73.50	56.97
TIMP3	Metalloproteinase inhibitor 3	24.44	13.49	3.32
VHL	Von Hippel–Lindau tumor suppressor	31.10	14.59	11.78
WIF1	Wnt inhibitory factor 1	98.14	82.56	60.68

## Discussion

Epigenetic alterations are commonly associated with carcinogenesis and metastasis (Wang et al., 2018a). Cancer metastasis is the major cause of treatment failure and mortality in women detected with cervical cancer. This suggests that the inhibition of metastasis serves a pivotal role in survival improvement, and hence can be exploited as a potential target for cancer treatment and prevention (Chatterjee et al., 2018). Additionally, epigenetic alteration in key metastatic genes is one of the reasons of metastasis (Chatterjee et al., 2018). The modifiable nature of epigenetics makes the epigenetic regulation an attractive target for cancer prevention and treatment (Busch et al., 2015; Kanwal et al., 2015). Currently available synthetic drugs direct at crucial epigenetic signature enzymes, for example, HDACs and DNMTs. Nevertheless, these synthetic drugs have shown many adverse side effects (Ho et al., 2013; Heerboth et al., 2014); therefore, it is crucial to probe the natural agents which are derived from plants that can regulate all cell processes including epigenetic mechanisms and can potentially reverse malignancy-associated epigenetic patterns (Shankar et al., 2016).

Earlier, studies on flavonoids targeting various types of cancer have demonstrated their anticancer effect by modulation of various molecular pathways involved in migration and epigenetics (Pal-Bhadra et al., 2012; Kanwal et al., 2016; Liskova et al., 2020). Considering the anticancer potential of plant flavonoids in view, the present study was carried out to explore the antiproliferative, antimigratory, and modulatory effects of chrysin on DNA methylation and histone modification on human cervical cancer (HeLa) cells. Previously, it was reported from the lab that chrysin inhibits the proliferation of HeLa cells in a dose- and time-dependent manner, induces apoptosis, and modulates various signaling pathways (Raina et al., 2021).

In the present study, 5 and 10 µM chrysin-treated HeLa cells showed reduced colony formation as 120 and 30 colonies were formed, respectively, after 48 h, whereas at 15 µM chrysin, there was no noticeable colony formation (Figure 1A and 1B). To understand chrysin-mediated antimigratory effects, the scratch-wound assay that depicted significant inhibition of cell migration was carried out. Chrysin treatment of HeLa cells at a conc. of 10 and 15 µM showed wound width increment by 8 and 17%, respectively, after 24 h incubation, whereas the wound width was increased by 14 and 25% after 48 h compared to the DMSO control, where complete wound closure was found after 48 h (Figure 2A and 2B). The trans-well assay also depicted the inhibition of migration at varying concentrations of chrysin (Figure 2C and Figure 2D). Hence, it can be inferred that chrysin at 10 and 15 μM at 48 h is a strong inhibitor of migration. Earlier studies from various research groups have also reported that chrysin bears antiproliferative and cytostatic effects and inhibits migration and invasion in various cancer cell lines (Kasala et al., 2015; Wang et al., 2018b).

Matrix metalloproteinases are important proteolytic enzymes involved in the cancer cell invasion process. Epithelial mesenchymal transition (EMT) increases cell migration, and the transforming growth factor pathway has an important role to play in epithelial mesenchymal transition; it induces EMT either by transforming growth factor-β (TGF-β)/SMAD pathway or *via* the non-SMAD pathway by activating the AKT/PI3K pathway, thus triggering migration (Wu et al., 2021; Xue et al., 2012). The downregulation of TGF-β/SMAD mediates reduction in the expression of MMPS and TWIST1 (Wu et al., 2021; Xue et al., 2012; Baruah et al., 2016). Cadherins are a class of type-1 transmembrane proteins that maintain the adhesion between cells, their loss lead to invasion and metastasis, and snails are their inhibitors. *MTA1* and *MTA2* are metastasis promoters, and their reduction leads to the inhibition of metastasis (Wu et al., 2021; Xue et al., 2012; Baruah et al., 2016). In the study, chrysin-mediated inhibition of migration was found to be well correlated with the downregulation of metalloproteases *MMP 9*, *MMP 2*, and *MMP 14* and their co-operators like *SMAD3*, *SMAD4*, *SNAIL1*, *SNAIL2*, *MTA1*, and *MTA2*, and upregulation of their inhibitors like *TIMP3*, *TIMP4*, and *CDH1*, hence endorsing the inhibitory effect of chrysin on migration. In addition, the downregulation of genes related to inflammation like *IL2*, *IL1A*, and *IL6* and oncogenes like *Fos*, *Jun*, *Myc*, *WNT1*, and *FOSL1* (Figure 3A and Table 1) was observed. Inflammation subsequent to viral infection is a power tool that accelerates cancer progression; hence, downregulation of inflammatory proteins aids in cancer prevention and treatment (Deivendran et al., 2014).

In the study, gene expression studies at mRNA levels were found to be consistent with their protein level expression, and various genes involved in inflammation and migration such as *HER1*, *HER2*, *Her3*, and *Her4*, and *MMPs* like *MMP 9*, *MMP 2*, *MMP 3*, *FOXC2*, *IL-2 Rα*, *IL-18 BPa*, *CXCL8/IL-8*, *CCL2/MCP-1*, *CL8/MCP-2*, *CCL7/MCP-3*, Mesothelin, *ICAM-1/CD54*, *MUC1*, Leptin, *M-CSF*, *H O-1/HMOX1*, *Kallikrein 5*, *MSP/MST1*, *Kallikrein 6*, *HIF-1α*, and *HNF-3β* were significantly downregulated against chrysin treatment, while upregulation of E-cadherins was observed at different chrysin concentrations (Figure 3B, 3C and Table 2). Our current findings are in line with the available reports wherein *in vitro/in vivo* models have shown that chrysin inhibits tumor metastasis by decreasing the expression of *MMP9* and *MMP2* as well as *COX-2* and *i-NOS*, and modulates the *PI3K/AKT* signaling pathway (Yang et al., 2014; Xia et al., 2015; Koosha et al., 2016; Zam and Khadour, 2017).

Furthermore, epigenetic modulations induced by chrysin were also analyzed in this study. Epigenetic modifications including aberrant DNA methylation and histone modifications interactions are crucial for controlling the operational activities of the genome by changing the chromatin structure, thereby leading to silencing of various tumor-suppressor genes (Guo et al., 2015; Chistiakov et al., 2017). DNMTs catalyze the transfer of the acetyl group onto 5′cytosine at promoter CpG island of TSGs. Hypermethylation of CpG islands in the promoter region of TSGs leads to silencing of these genes (Ali Khan et al., 2015). DNMTs are found to be upregulated in cervical cancer cells, and their expression levels are correlated to disease progression (Piyathilake et al., 2017; Charostad et al., 2019). The analysis of biochemical activity of DNMTs after chrysin treatment was performed, and it was observed that chrysin decreased the biochemical activity of DNMTs in a dose-dependent manner, and it was reduced by 35, 53.5, and 61.2% at 5, 10, and 15 µM chrysin treatment, respectively (Figure 4A).

Furthermore, the downregulation of DNMT activity against chrysin treatment was verified by downregulation of *DNMT1*, *DNMT3A*, and *DNMT3B* in a dose-dependent manner at transcript levels (Figure 5A and Table 3). Various flavonoids have attracted attention because of their chemopreventive and antitumor effects including chrysin, luteolin, and apigenin, and are known to inhibit DNMTs and histone methyl transferases (Busch et al., 2015; Kanwal et al., 2016). The decrease in DNMT expression after chrysin treatment was well correlated with the decrease in global DNA methylation (Figure 6A) and modulation pattern of CpG promoter methylation of TSGs (*APC*, *BRCA1*, *FHIT*, *CDH1*, *CDH13*, *MGMT*, *MLH1*, *GSTP1*, *TIMP3*, *RARB*, *RASSIF1*, *SOCS1*, *PTEN*, *VHL*, and *WIFI*) (Figure 6B) after chrysin treatment of 10 and 15 µM for 48 h. This study revealed that chrysin treatment downregulated global DNA methylation levels by decreasing to 61, 44, and 30% at 5, 10, and 15 µM chrysin treatment, respectively, for 48 h compared to DMSO controls (Figure 6A). Hypermethylation of TSGs leads to silencing of these genes and has been found to be well correlated with the overexpression of various DNMTs in cervical cancer (Guo et al., 2015; Jiménez–wences et al., 2014). Interestingly, this is the very first time it has been reported that chrysin treatment significantly decreases the methylation levels at the promoter region of several TSGs *viz APC*, *CDH1*, *CDH13*, *BRCA1*, *CDKN2A*, *DAPK1*, *FHIT*, *GSTP1*, *MGMT*, *MLH1*, *PTEN*, *RARB*, *RASSF1*, *SOCS1*, *TIMP3*, and *WIFI* (Figure 6B and Table 4 *)*, which are found to be hypermethylated in many cancers and have critical roles to play in various cellular processes (Mukherjee et al., 2015). Hypermethylation of *PTEN* and *RASSIF1* is a common feature in cervical cancers. *PTEN* has an important role in cell migration and proliferation, and inhibits migration by being the antagonist of MMPs (Salimi Sartakhti et al., 2017), whereas reduced RASSIF1 quenches cell death by the receptor mode. VHL is important for stabilization of HIF1 and HIF2, and methylation of other TSGs like RARB and FHIT leads to uncontrolled proliferation; GSTP1 is involved in detoxification of harmful compounds, and MGMT is important for DNA repair (Mukherjee et al., 2015). A reduction of CpG methylation at the abovementioned gene loci can be correlated to reactivation of these genes at the transcription level; chrysin treatment upregulated the expression of *PTEN*, *CDH1*, *TP53*, *FHIT*, *RASSIFI*, *SOCS1*, *RARB*, *TIMP3*, and *TIMP4* (Figure 3A) in our study. Several polyphenols including chrysin and luteolin have shown modulation of methylation, and thus reactivation of the silenced TSGs (Aggarwal et al., 2015; Ali Khan et al., 2015; Busch et al., 2015; Kanwal et al., 2016; Carlos-Reyes et al., 2019).

Apart from DNA modification, histone modifications like histone acetylation and histone methylation influence the expression of various genes that have an important role in cancer cell proliferation and migration (Chakravarthy et al., 2005; Dueñas–González et al., 2005; Soto et al., 2017). HDACs deacetylate histone and non-histone proteins such as TP53, rendering them non-functional (Chakrabarti et al., 2015). Overexpression of HDAC1, HDAC2, HDAC3, and HDAC6 has been reported in different cancers (Kogan et al., 2017; Lin et al., 2009; Huang et al., 2005; Zhang et al., 2016; Ahn and Yoon, 2017). HDAC overexpression together with DNA methylation and other histone modifications silences tumor-suppressor genes (Rose and Klose, 2014; Jenuwein and Allis, 2001). In our study, chrysin was found to decrease the expression of various HDACs (1, 2, 3, 11, and 4), HAT1, EHM2, AURKA, AURKB, PRMT 8, ASH1l, USP21, and USP22 at the transcript level, and increased the expression of ESCO2 and CIITA in a dose-dependent manner (RQs are given in Table 3 and Figure 5A). Chrysin treatment decreased the HDAC activity in a dose-dependent manner; HDAC activity decreased by 30, 36, and 42% after 5, 10, and 15 µM chrysin treatment, respectively (Figure 4B). This was further endorsed by decrease in the expression at the transcript level of HDACs 1, 2, 3, 4, and 11 in a significant manner at 10 and 15 µM chrysin treatment for 48 h (Figure 5A). HAT1 is upregulated in cervical cancer and is responsible for the induction of colony formation (Kedhari Sundaram et al., 2019b). Chrysin downregulated the activity of HAT by 22, 34, and 52% at 5, 10, and 15 µM chrysin, respectively (Figure 4C). It also reduced the expression of HAT in a dose-dependent manner at the transcript level with RQ of 0.31 at 15 µM (Figure 5A) and thus correlated with complete inhibition of colony formation. Similar results were reported by other researchers who observed inhibition of HDAC two and eight and upregulation of H3acK14, H4acK12, and H4acK16, and decrease in H3me2K9 methylation in different cell lines (Pal-Bhadra et al., 2012; Sun et al., 2012).

CIITA and ESCO2 were up-regulated after chrysin treatment (Figure 5A). CIITA positively regulates the expression of class II major histocompatibility complex and is often found to be methylated in cancer cells (Ramia et al., 2019). ESCO2 histone acetyltransferase curbs MMP2 and also encourages apoptosis in cancer cells (Guo et al., 2018). Trimethylation of lysine 9 and 27 of histone 3 (H3K9 and H3K27) at the promoter region is related to reduced TSG expression (Trievel, 2004; Daniel et al., 2005; Lachner et al., 2001). These marks are found to be overactive in cervical cancer (Chen et al., 2017). Remarkably, in the current study, chrysin downregulated all mono, di, and trimethylation marks at H3K9 (Figure 5B), and this was further verified by the assessment of H3K9 methyltransferase activity after incubation of HeLa cells with chrysin. Also, it was found that H3K9 HMT activity was significantly reduced by 25, 38, and 45% against 5, 10, and 15 µM chrysin treatment (Figure 4D). Moreover, chrysin also downregulated EHM2 expression (Figure 5A), which is responsible for methylation of H3K9. All the methylation marks at H3K27, H3K36, H3K79, and H3K4 were downregulated after chrysin treatment (Figure 5B). H3K4 mono and demethylation are related to transcription activation (Chang and Yu, 2016b). H4 acetylation marks such as H4K5, H4K8, H4K12, and H4k16 were also reduced by chrysin (Figure 5C). This is in line with the previously published report on a flavone luteolin, wherein it blocks the acetylation of histone H4 and controls the activity of c-FOS, p21, and other genes related to cell cycle control (Izzo et al., 2020). Thus, it can be suggested that chrysin is a potent inhibitor of DNA methyl transferases and histone methyltransferases, and thus modulates the methylation of TSGs.

The overexpression of any one of the EMT inducers such as Twist TGF-β1 or Snail upregulates FOXC2 expression and can lead to the initiation of EMT (Mani et al., 2007). In fact, a significant cadherin switch from E-cadherin to N-cadherin is expressed in cancer progression (Kouzarides, 2007). VE-cadherin, another cadherin, mediates cell-to-cell bonding by holding the catenin in between, which in turn connects the actin cytoskeleton of the cells. Both E cadherin and VE-cadherin are down-regulated in cancer progression (Ramis-Conde et al., 2009). It was observed that chrysin (10 and 15 µM for 48 h) decreased the expression of *SNAIL*, *TWIST* (Figure 3A), and FOXC2 (Figure 3B and 3C), and increased the expression of E cadherin (Figure 3B). The suppressor of cytokine signaling 1 (SOCS1) is a tumor-suppressor gene and suppresses cytokine signaling and destroys the HPV E7 protein. SOCS1 is hypermethylated in cervical cancer and renewal of its expression upsurges Rb protein thereby inhibits cell proliferation (Kamio et al., 2004; Sobti et al., 2011). A decrease in E cadherin can be linked to WNT signaling which prevents phosphorylation of SNAIL, allowing it to accumulate and repress cadherin (Loh et al., 2019). Based upon the findings from the present study, it can be proposed that the restoration of transcription in the tumor-suppressor genes plays a crucial role in the anticancer potential of chrysin against HeLa cells, as it can directly influence cell proliferation and cell migration. Our current results are based on the chrysin efficacy on HeLa cells, but can further be extrapolated on other cell lines and animal models.

## Conclusion

Chrysin appears to be a promising natural chemopreventive agent which is cytotoxic to cancer cells and inhibits migration, diminishes CpG promoter methylation of TSG modulates, and causes re-expression of TSG, and downregulation of genes related to migration and inflammation. Hence, chrysin can be exploited for further use at a clinical setup after experimental validation and checking its pharmacokinetic properties involving human subjects.

## Data Availability

The original contributions presented in the study are included in the article, further inquiries can be directed to the corresponding authors.

## References

[B1] Aggarwal R. Jha M. Shrivastava A. Jha A. K. (2015). Natural Compounds: Role in Reversal of Epigenetic Changes. Biochem. Mosc. 80, 972–989. 10.1134/S0006297915080027 26547065

[B2] Ahn M.-Y. Yoon J.-H. (2017). Histone Deacetylase 7 Silencing Induces Apoptosis and Autophagy in Salivary Mucoepidermoid Carcinoma Cells. J. Oral Pathol. Med. 46, 276–283. 10.1111/jop.12560 28178760

[B3] Ali Khan M. Kedhari Sundaram M. Hamza A. Quraishi U. Gunasekera D. Ramesh L. (2015). Sulforaphane Reverses the Expression of Various Tumor Suppressor Genes by Targeting DNMT3B and HDAC1 in Human Cervical Cancer Cells. Evidence-Based Complement. Altern. Med. 2015, 1–12. 10.1155/2015/412149 PMC4487331 26161119

[B4] Andrijauskaite K. Morris J. Wargovich M. J. (2019). “Natural Anticancer Agents,” in Epigenetics of Cancer Prevention (Elsevier), 49–73. 10.1016/b978-0-12-812494-9.00003-2

[B5] Baruah M. M. Khandwekar A. P. Sharma N. (2016). Quercetin Modulates Wnt Signaling Components in Prostate Cancer Cell Line by Inhibiting Cell Viability, Migration, and Metastases. Tumor Biol. 37, 14025–14034. 10.1007/s13277-016-5277-6 27495232

[B6] Busch C. Burkard M. Leischner C. Lauer U. M. Frank J. Venturelli S. (2015). Epigenetic Activities of Flavonoids in the Prevention and Treatment of Cancer. Clin. Epigenet. 7, 64. 10.1186/s13148-015-0095-z PMC4497414 26161152

[B7] Carlos-Reyes Á. López-González J. S. Meneses-Flores M. Gallardo-Rincón D. Ruíz-García E. Marchat L. A. (2019). Dietary Compounds as Epigenetic Modulating Agents in Cancer. Front. Genet. 10, 1–14. 10.3389/fgene.2019.00079 30881375 PMC6406035

[B8] Chakrabarti A. Oehme I. Witt O. Oliveira G. Sippl W. Romier C. (2015). HDAC8: a Multifaceted Target for Therapeutic Interventions. Trends Pharmacol. Sci. 36, 481–492. 10.1016/j.tips.2015.04.013 26013035

[B9] Chakravarthy S. Park Y.-J. Chodaparambil J. Edayathumangalam R. S. Luger K. (2005). Structure and Dynamic Properties of Nucleosome Core Particles. FEBS Lett. 579, 895–898. 10.1016/j.febslet.2004.11.030 15680970

[B10] Chang L.-C. Yu Y.-L. (2016). Dietary Components as Epigenetic-Regulating Agents against Cancer. BioMed 6, 9–16. 10.7603/s40681-016-0002-8 PMC4752550 26872811

[B11] Chang L. C. Yu Y. L. (2016). Dietary Components as Epigenetic-Regulating Agents against Cancer. Biomedicine (Taipei) 6, 2–8. 10.7603/s40681-016-0002-8 26872811 PMC4752550

[B12] Charostad J. Astani A. Goudarzi H. Faghihloo E. (2019). DNA Methyltransferases in Virus-Associated Cancers. Rev. Med. Virol. 29, e2022. 10.1002/rmv.2022 30511446

[B13] Chatterjee A. Rodger E. J. Eccles M. R. (2018). Epigenetic Drivers of Tumourigenesis and Cancer Metastasis. Semin. Cancer Biol. 51, 149–159. 10.1016/j.semcancer.2017.08.004 28807546

[B14] Chen R.-J. Shun C.-T. Yen M.-L. Chou C.-H. Lin M.-C. (2017). Methyltransferase G9a Promotes Cervical Cancer Angiogenesis and Decreases Patient Survival. Oncotarget 8, 62081–62098. 10.18632/oncotarget.19060 28977928 PMC5617488

[B15] Chistiakov D. A. D. Myasoedova V. A. V. Orekhov A. N. Bobryshev Y. V. (2017). Epigenetically Active Drugs Inhibiting DNA Methylation and Histone Deacetylation. Cpd 23, 1167–1174. 10.2174/1381612822666161021110827 27774908

[B16] Crowley L. C. Marfell B. J. Scott A. P. Boughaba J. A. Chojnowski G. Christensen M. E. (2016). Dead Cert: Measuring Cell Death. Cold Spring Harb. Protoc. 2016, pdb.top070318–1072. 10.1101/pdb.top070318 27934691

[B17] Daniel J. A. Pray-Grant M. G. Grant P. A. (2005). Effector Proteins for Methylated Histones: an Expanding Family. Cell Cycle 4, 919. 10.4161/cc.4.7.1824 15970672

[B18] Deivendran S. Marzook K. H. Radhakrishna Pillai M. (2014). The Role of Inflammation in Cervical Cancer. Adv. Exp. Med. Biol. 816, 377–399. 10.1007/978-3-0348-0837-8_15 24818731

[B19] Dueñas-González A. Lizano M. Candelaria M. Cetina L. Arce C. Cervera E. (2005). Epigenetics of Cervical Cancer. An Overview and Therapeutic Perspectives. Mol. Cancer 4, 1–24. 10.1186/1476-4598-4-38 16248899 PMC1291396

[B20] Ganai S. A. Sheikh F. A. Baba Z. A. Mir M. A. Mantoo M. A. Yatoo M. A. (2021). Anticancer Activity of the Plant Flavonoid Luteolin against Preclinical Models of Various Cancers and Insights on Different Signalling Mechanisms Modulated. Phytotherapy Res. 35, 3509–3532. 10.1002/ptr.7044 33580629

[B21] Ganai S. A. Sheikh F. A. Baba Z. A. (2021). Plant Flavone Chrysin as an Emerging Histone Deacetylase Inhibitor for Prosperous Epigenetic‐based Anticancer Therapy. Phytotherapy Res. 35, 823–834. 10.1002/ptr.6869 32930436

[B22] Guo X.-B. Huang B. Pan Y.-H. Su S.-G. Li Y. (2018). ESCO2 Inhibits Tumor Metastasis via Transcriptionally Repressing MMP2 in Colorectal Cancer. Cmar 10, 6157–6166. 10.2147/cmar.s181265 PMC6257866 30538563

[B23] Guo Y. Su Z.-Y. Kong A.-N. T. (2015). Current Perspectives on Epigenetic Modifications by Dietary Chemopreventive and Herbal Phytochemicals. Curr. Pharmacol. Rep. 1, 245–257. 10.1007/s40495-015-0023-0 26328267 PMC4552355

[B24] Hatzimichael E. Crook T. (2013). Cancer Epigenetics: New Therapies and New Challenges. J. Drug Deliv. 2013, 529312. 10.1155/2013/529312 23533770 PMC3600296

[B25] Heerboth S. Lapinska K. Snyder N. Leary M. Rollinson S. Sarkar S. (2014). Use of Epigenetic Drugs in Disease: An Overview. Genet. Epigenet. 6, GEG.S12270–19. 10.4137/GeG.s12270 PMC4251063 25512710

[B26] Herranz M. Esteller M. (2007). “DNA Methylation and Histone Modifications in Patients with Cancer,” in Target Discovery and Validation Reviews and Protocols (Springer), 25–62. 10.1385/1-59745-208-4:25 17172706

[B27] Herranz M. Esteller M. (2007). DNA Methylation and Histone Modifications in Patients with Cancer: Potential Prognostic and Therapeutic Targets. Methods Mol. Biol. 361, 25–62. 10.1385/1-59745-208-4:25 17172706

[B28] Ho A. S. Turcan S. Chan T. A. (2013). Epigenetic Therapy: Use of Agents Targeting Deacetylation and Methylation in Cancer Management. Onco Targets Ther. 6, 223–232. 10.2147/OTT.S34680 23569385 PMC3615839

[B29] Huang B. H. Laban M. Leung C. H.-W. Lee L. Lee C. K. Salto-Tellez M. (2005). Inhibition of Histone Deacetylase 2 Increases Apoptosis and p21Cip1/WAF1 Expression, Independent of Histone Deacetylase 1. Cell Death Differ. 12, 395–404. 10.1038/sj.cdd.4401567 15665816

[B30] Huang J. Plass C. Gerhauser C. (2011). Cancer Chemoprevention by Targeting the Epigenome. Cdt 12, 1925–1956. 10.2174/138945011798184155 21158707

[B31] Izzo S. Naponelli V. Bettuzzi S. (2020). Flavonoids as Epigenetic Modulators for Prostate Cancer Prevention. Nutrients 12, 1010–1024. 10.3390/nu12041010 PMC7231128 32268584

[B32] Jenuwein T. Allis C. D. (2001). Translating the Histone Code. Science 293, 1074–1080. 10.1126/science.1063127 11498575

[B33] Jiménez-wences H. Peralta-Zaragoza O. Fernández-tilapa G. (2014). Human Papilloma Virus, DNA Methylation and microRNA Expression in Cervical Cancer (Review). Oncol. Rep. 31, 2467–2476. 10.3892/or.2014.3142 24737381 PMC4055305

[B34] Kai L. Samuel S. K. Levenson A. S. (2010). Resveratrol Enhances P53 Acetylation and Apoptosis in Prostate Cancer by Inhibiting MTA1/NuRD Complex. Int. J. Cancer 126, 1538–1548. 10.1002/ijc.24928 19810103

[B35] Kamio M. Yoshida T. Ogata H. Douchi T. Nagata Y. Inoue M. (2004). SOC1 Inhibits HPV-E7-Mediated Transformation by Inducing Degradation of E7 Protein. Oncogene 23, 3107–3115. 10.1038/sj.onc.1207453 15021916

[B36] Kanwal R. Gupta K. Gupta S. (2015). “Aberrant DNA Methylation Is One of the Most Important Epigenetic Modifications in Cancer Cells and it Is Also Associated with Histone Modifications and Their Interaction of Is Crucial to Regulate the Functioning of the Genome by Changing Chromatin Archite,” in Cancer Epigenetics (Springer), 3–25.

[B37] Kanwal R. Datt M. Liu X. Gupta S. (2016). Dietary Flavones as Dual Inhibitors of DNA Methyltransferases and Histone Methyltransferases. PLoS One 11, e0162956. 10.1371/journal.pone.0162956 27658199 PMC5033486

[B38] Kasala E. R. Bodduluru L. N. Madana R. M. V A. K. Gogoi R. Barua C. C. (2015). Chemopreventive and Therapeutic Potential of Chrysin in Cancer: Mechanistic Perspectives. Toxicol. Lett. 233, 214–225. 10.1016/j.toxlet.2015.01.008 25596314

[B39] Kedhari Sundaram M. Raina R. Afroze N. Bajbouj K. Hamad M. Haque S. (2019). Quercetin Modulates Signaling Pathways and Induces Apoptosis in Cervical Cancer Cells. Biosci. Rep. 39. 10.1042/BSR20190720 PMC6692570 31366565

[B40] Kedhari Sundaram M. Hussain A. Haque S. Raina R. Afroze N. (2019). Quercetin Modifies 5′CpG Promoter Methylation and Reactivates Various Tumor Suppressor Genes by Modulating Epigenetic marks in Human Cervical Cancer Cells. J. Cel. Biochem. 120, 18357–18369. 10.1002/jcb.29147 31172592

[B41] Khoo B. Y. Chua S. L. Balaram P. (2010). Apoptotic Effects of Chrysin in Human Cancer Cell Lines. Ijms 11, 2188–2199. 10.3390/ijms11052188 20559509 PMC2885101

[B42] Kim S. O. Kim M. R. (2013). (-)-Epigallocatechin 3-gallate Inhibits Invasion by Inducing the Expression of Raf Kinase Inhibitor Protein in AsPC-1 Human Pancreatic Adenocarcinoma Cells through the Modulation of Histone Deacetylase Activity. Int. J. Oncol. 42, 349–358. 10.3892/ijo.2012.1686 23135610

[B43] Kogan E. A. Unanyan A. L. Kadyrova A. E. Demura T. A. Sidorova I. S. Faizullin R. I. (2017). Immunohistochemical Analysis of Epigenetic Markers in Cervical Pathologies Associated with Human Papillomavirus Infection. BioNanoSci. 7, 284–287. 10.1007/s12668-016-0339-1

[B44] Koosha S. Alshawsh M. A. Looi C. Y. Seyedan A. Mohamed Z. (2016). An Association Map on the Effect of Flavonoids on the Signaling Pathways in Colorectal Cancer. Int. J. Med. Sci. 13, 374–385. 10.7150/ijms.14485 27226778 PMC4879672

[B45] Kouzarides T. (2007). Chromatin Modifications and Their Function. Cell 128, 693–705. 10.1016/j.cell.2007.02.005 17320507

[B46] Lachner M. O'Carroll D. Rea S. Mechtler K. Jenuwein T. (2001). Methylation of Histone H3 Lysine 9 Creates a Binding Site for HP1 Proteins. Nature 410, 116–120. 10.1038/35065132 11242053

[B47] Li L-L. Wang S-S. (2017). DNA Methyltransferase (DNMTs) Expression in Cervical Cancer Tissues and its Relationship with HPV Infection and Tumor Malignancy. J. Hainan Med. Univ. 23, 136–139.

[B48] Lim W. Ryu S. Bazer F. W. Kim S.-M. Song G. (2018). Chrysin Attenuates Progression of Ovarian Cancer Cells by Regulating Signaling Cascades and Mitochondrial Dysfunction. J. Cel. Physiol. 233, 3129–3140. 10.1002/jcp.26150 28816359

[B49] Lin Z. Bazzaro M. Wang M.-C. Chan K. C. Peng S. Roden R. B. S. (2009). Combination of Proteasome and HDAC Inhibitors for Uterine Cervical Cancer Treatment. Clin. Cancer Res. 15, 570–577. 10.1158/1078-0432.CCR-08-1813 19147762 PMC2714480

[B50] Liskova A. Koklesova L. Samec M. Smejkal K. Samuel S. M. Varghese E. (2020). Flavonoids in Cancer Metastasis. Cancers 12, 1498. 10.3390/cancers12061498 PMC7352928 32521759

[B51] Loh C.-Y. Chai J. Tang T. Wong W. Sethi G. Shanmugam M. (2019). The E-Cadherin and N-Cadherin Switch in Epithelial-To-Mesenchymal Transition: Signaling, Therapeutic Implications, and Challenges. Cells 8, 1118. 10.3390/cells8101118 PMC6830116 31547193

[B52] Mani S. A. Yang J. Brooks M. Schwaninger G. Zhou A. Miura N. (2007). Mesenchyme Forkhead 1 (FOXC2) Plays a Key Role in Metastasis and Is Associated with Aggressive Basal-like Breast Cancers. Proc. Natl. Acad. Sci. 104, 10069–10074. 10.1073/pnas.0703900104 17537911 PMC1891217

[B53] McLaughlin-Drubin M. E. Crum C. P. Münger K. (2011). Human Papillomavirus E7 Oncoprotein Induces KDM6A and KDM6B Histone Demethylase Expression and Causes Epigenetic Reprogramming. Proc. Natl. Acad. Sci. USA 108, 2130–2135. 10.1073/pnas.1009933108 21245294 PMC3033314

[B54] Mocanu M.-M. Nagy P. Szöllősi J. (2015). Chemoprevention of Breast Cancer by Dietary Polyphenols. Molecules 20, 22578–22620. 10.3390/molecules201219864 26694341 PMC6332464

[B55] Mukherjee N. Kumar A. P. Ghosh R. (2015). DNA Methylation and Flavonoids in Genitourinary Cancers. Curr. Pharmacol. Rep. 1, 112–120. 10.1007/s40495-014-0004-8 26005633 PMC4437245

[B56] Ong T. P. Moreno F. S. Ross S. A. (2011). Targeting the Epigenome with Bioactive Food Components for Cancer Prevention. J. Nutrigenet. Nutrigenomics 4, 275–292. 10.1159/000334585 22353664 PMC3388269

[B57] Pal-Bhadra M. Ramaiah M. J. Reddy T. L. Krishnan A. Pushpavalli S. Babu K. S. (2012). Plant HDAC Inhibitor Chrysin Arrest Cell Growth and Induce P21 WAF1 by Altering Chromatin of STAT Response Element in A375 Cells. BMC Cancer 12, 180. 10.1186/1471-2407-12-180 22591439 PMC3407000

[B58] Paredes-Gonzalez X. Fuentes F. Su Z.-Y. Kong A.-N. T. (2014). Apigenin Reactivates Nrf2 Anti-oxidative Stress Signaling in Mouse Skin Epidermal JB6 P + Cells through Epigenetics Modifications. AAPS J. 16, 727–735. 10.1208/s12248-014-9613-8 24830944 PMC4070251

[B59] Piyathilake C. Badiga S. Borak S. Weragoda J. Bae S. Matthews R. (2017). A Higher Degree of Expression of DNA Methyl Transferase 1 in Cervical Cancer Is Associated with Poor Survival Outcome. Ijwh 9, 413–420. 10.2147/IJWH.S133441 PMC5476577 28652820

[B60] Rahman M. S. Jamil H. M. Akhtar N. Islam R. Abdul-Awal S. M. Rana M. M. (2016). Cancer Epigenetics and Epigenetical Therapy. J. Exp. Integr. Med. 6, 1. 10.5455/jeim.270616.rw.016

[B61] Raina R. Afroze N. Kedhari Sundaram M. Haque S. Bajbouj K. Hamad M. (2021). Chrysin Inhibits Propagation of HeLa Cells by Attenuating Cell Survival and Inducing Apoptotic Pathways. Eur. Rev. Med. Pharmacol. Sci. 25, 2206–2220. 10.26355/eurrev_202103_25253 33755959

[B62] Ramia E. Chiaravalli A. M. Bou Nasser Eddine F. F. Tedeschi A. Sessa F. Accolla R. S. (2019). CIITA-related Block of HLA Class II Expression, Upregulation of HLA Class I, and Heterogeneous Expression of Immune Checkpoints in Hepatocarcinomas: Implications for New Therapeutic Approaches. Oncoimmunology 8, 1548243. 10.1080/2162402X.2018.1548243 30723578 PMC6350839

[B63] Ramis-Conde I. Chaplain M. A. Anderson A. R. Drasdo D. (2009). Multi-scale Modelling of Cancer Cell Intravasation: the Role of Cadherins in Metastasis. Phys. Biol. 6, 016008. 10.1088/1478-3975/6/1/016008 19321920

[B64] Rose N. R. Klose R. J. (2014). Understanding the Relationship between DNA Methylation and Histone Lysine Methylation. Biochim. Biophys. Acta (Bba) - Gene Regul. Mech. 1839, 1362–1372. 10.1016/j.bbagrm.2014.02.007 PMC4316174 24560929

[B65] Ryu S. Lim W. Bazer F. W. Song G. (2017). Chrysin Induces Death of Prostate Cancer Cells by Inducing ROS and ER Stress. J. Cel. Physiol. 232, 3786–3797. 10.1002/jcp.25861 28213961

[B66] Salimi Sartakhti J. Manshaei M. H. Sadeghi M. (2017). MMP-TIMP Interactions in Cancer Invasion: An Evolutionary Game-Theoretical Framework. J. Theor. Biol. 412, 17–26. 10.1016/j.jtbi.2016.09.019 27670802

[B67] Shankar E. Kanwal R. Candamo M. Gupta S. (2016). Dietary Phytochemicals as Epigenetic Modifiers in Cancer: Promise and Challenges. Semin. Cancer Biol. 40-41, 82–99. 10.1016/j.semcancer.2016.04.002 27117759 PMC5067170

[B68] Sharma S. Kelly T. K. Jones P. A. (2009). Epigenetics in Cancer. Carcinogenesis 31, 27–36. 10.1093/carcin/bgp220 19752007 PMC2802667

[B69] Sobti R. C. Singh N. Hussain S. Suri V. Nijhawan R. Bharti A. C. (2011). Aberrant Promoter Methylation and Loss of Suppressor of Cytokine Signalling-1 Gene Expression in the Development of Uterine Cervical Carcinogenesis. Cell Oncol. 34, 533–543. 10.1007/s13402-011-0056-2 PMC12995090 21935712

[B70] Soto D. Song C. McLaughlin-Drubin M. E. (2017). Epigenetic Alterations in Human Papillomavirus-Associated Cancers. Viruses 9, 248. 10.3390/v9090248 PMC5618014 28862667

[B71] Sun L.-P. Chen A.-L. Hung H.-C. Chien Y.-H. Huang J.-S. Huang C.-Y. (2012). Chrysin: a Histone Deacetylase 8 Inhibitor with Anticancer Activity and a Suitable Candidate for the Standardization of Chinese Propolis. J. Agric. Food Chem. 60, 11748–11758. 10.1021/jf303261r 23134323

[B72] Trievel R. C. (2004). Structure and Function of Histone Methyltransferases. Crit. Rev. Eukaryot. Gene Expr. 14, 147–170. 10.1615/CritRevEukaryotGeneExpr.v14.i3.10 15248813

[B73] Wang J. Wang H. Sun K. Wang X. Pan H. Zhu J. (2018). Chrysin Suppresses Proliferation, Migration, and Invasion in Glioblastoma Cell Lines via Mediating the ERK/Nrf2 Signaling Pathway. Dddt 12, 721–733. 10.2147/dddt.s160020 29662304 PMC5892952

[B74] Wang X. Jiang Z. An J. Mao X. Lin F. Sun P. (2018). Effect of a Synthetic Inhibitor of Urokinase Plasminogen Activator on the Migration and Invasion of Human Cervical Cancer Cells *In Vitro* . Mol. Med. Rep. 17, 4273–4280. 10.3892/mmr.2018.8414 29328476 PMC5802199

[B75] Wu D. Zhao B. Song Y. Chi X. Fu H. Guan T. (2021). Nogo-B Receptor Is Required for Stabilizing TGF-β Type Ireceptor and Promotes the TGF-Β1-Induced Epithelial-To-Mesenchymal Transition of Non-small Cell Lung Cancer. J. Cancer 12, 717–725. 10.7150/jca.50483 33403029 PMC7778533

[B76] Xia Y. Lian S. Khoi P. N. Yoon H. J. Joo Y. E. Chay K. O. (2015). Chrysin Inhibits Tumor Promoter-Induced MMP-9 Expression by Blocking AP-1 via Suppression of ERK and JNK Pathways in Gastric Cancer Cells. PLoS One 10, e0124007. 10.1371/journal.pone.0124007 25875631 PMC4398353

[B77] Xue G. Restuccia D. F. Lan Q. Hynx D. Dirnhofer S. Hess D. (2012). Akt/PKB-Mediated Phosphorylation of Twist1 Promotes Tumor Metastasis via Mediating Cross-Talk between PI3K/Akt and TGF-β Signaling Axes. Cancer Discov. 2, 248–259. 10.1158/2159-8290.cd-11-0270 22585995

[B78] Yan W. Wu T. H. Y. Leung S. S. Y. To K. K. W. (2020). Flavonoids Potentiated Anticancer Activity of Cisplatin in Non-small Cell Lung Cancer Cells *In Vitro* by Inhibiting Histone Deacetylases. Life Sci. 258, 118211. 10.1016/j.lfs.2020.118211 32768576

[B79] Yang B. Huang J. Xiang T. Yin X. Luo X. Huang J. (2014). Chrysin Inhibits Metastatic Potential of Human Triple-Negative Breast Cancer Cells by Modulating Matrix Metalloproteinase-10, Epithelial to Mesenchymal Transition, and PI3K/Akt Signaling Pathway. J. Appl. Toxicol. 34, 105–112. 10.1002/jat.2941 24122885

[B80] You J. S. Jones P. A. (2012). Cancer Genetics and Epigenetics: Two Sides of the Same Coin? Cancer Cell 22, 9–20. 10.1016/j.ccr.2012.06.008 22789535 PMC3396881

[B81] Zam W. Khadour A. (2017). Impact of Phytochemicals and Dietary Patterns on Epigenome and Cancer. Nutr. Cancer 69, 184–200. 10.1080/01635581.2017.1263746 28094554

[B82] Zhang L. Yuan C. Wang Y. Zhao S. (2016). Histone Deacetylases 3 ( HDAC3 ) Is Highly Expressed in Cervical Cancer and Inhibited by siRNA. Int. J. Clin. Exp. Pathol. 9, 3600–3605.

